# Comparison of serum markers for muscle damage, surgical blood loss, postoperative recovery, and surgical site pain after extreme lateral interbody fusion with percutaneous pedicle screws or traditional open posterior lumbar interbody fusion

**DOI:** 10.1186/s12891-017-1775-y

**Published:** 2017-10-16

**Authors:** Tetsuro Ohba, Shigeto Ebata, Hirotaka Haro

**Affiliations:** Department of Orthopaedics, University of Yamanashi, 1110 Shimokato, Chuo, Yamanashi 409-3898 Japan

**Keywords:** Lumbar degenerative spondylolisthesis, Extreme lateral interbody fusion, Percutaneous pedicle screws, Minimally invasive surgery, Muscle damage, Low back pain

## Abstract

**Background:**

The benefits of extreme lateral interbody fusion (XLIF) as a minimally invasive lumbar spinal fusion treatment for lumbar degenerative spondylolisthesis have been unclear. We sought to evaluate the invasiveness and tolerability of XLIF with percutaneous pedicle screws (PPS) compared with traditional open posterior lumbar interbody fusion (PLIF).

**Methods:**

Fifty-six consecutive patients underwent open PLIF and 46 consecutive patients underwent single-staged treatment with XLIF with posterior PPS fixation for degenerative lumbar spondylolisthesis, and were followed up for a minimum of 1 year. We analyzed postoperative serum makers for muscle damage and inflammation, postoperative surgical pain, and performance status. A Roland–Morris Disability Questionnaire (RDQ) and Oswestry Disability Index (ODI) were obtained at the time of hospital admission and 1 year after surgery.

**Results:**

Intraoperative blood loss (51 ± 41 ml in the XLIF/PPS group and 206 ± 191 ml in the PLIF group), postoperative WBC counts and serum CRP levels in the XLIF/PPS group were significantly lower than in the PLIF group. Postoperative serum CK levels were significantly lower in the XLIF/PPS group on postoperative days 4 and 7. Postoperative recovery of performance was significantly greater in the XLIF/PPS group than in the PLIF group from postoperative days 2 to 7. ODI and visual analog scale (VAS) score (lumbar) 1 year after surgery were significantly lower in the XLIF/PPS group compared with the PLIF group.

**Conclusions:**

The XLIF/PPS procedure is advantageous to minimize blood loss and muscle damage, with consequent earlier recovery of daily activities and reduced incidence of low back pain after surgery than with the open PLIF procedure.

## Background

Spinal fusion is a surgical procedure used to fuse two or more vertebrae and to stabilize unstable spine segments. Lumbar spinal fusion surgery has been widely used to manage the pain and neurological symptoms in patients with low back pain (LBP) [1]. Traditional open posterior approaches for fusion and supplemental internal fixation that require extensive dissection of paraspinal musculature can result in permanent erector spinae denervation, loss of function, and late onset of spinal instability [2, 3]. Open lumbar spine surgeries are often accompanied by surgical site pain compared with minimally invasive techniques [4, 5].

Alternatively, more modern, less invasive approaches for lumbar interbody fusion have gained in popularity, one such approach being the mini-open lateral transpsoas approach (XLIF, NuVasive, San Diego, CA, USA) [6]. Benefits of the lateral approach include the preservation of back muscle, and bony and ligamentous structures, and it also allows for the placement of an intervertebral cage. In addition, the current procedure results in correction of spondylolisthesis and rotatory deformity, and indirect nerve decompression by ligamentotaxis force. These advantages may result in less surgical pain and quicker recovery than achieved in traditional approaches [7]. The validity of minimally invasive lumbar interbody fusions with percutaneous pedicle screws (PPS) has been described [8, 9].

By contrast, a comparatively high complication rate of XLIF including postoperative thigh symptoms (range 1–60.1%) has been reported [10]. A recent review concluded there is insufficient evidence for the comparative effectiveness of XLIF compared with traditional posterior lumbar interbody fusion (PLIF) [11, 12]. To evaluate the invasiveness and tolerability of XLIF with PPS compared with PLIF, we evaluated serum markers of muscle damage and inflammation, surgical pain, surgical blood loss, and postoperative recovery of activities of daily living (performance status score) for XLIF with PPS compared with traditional open PLIF surgery.

## Methods

### Patient group and surgical techniques

Patients were candidates for surgery if fusion was indicated because of degenerative lumbar spondylolisthesis and if a full course of conservative care, in particular, drug and brace treatments, had been exhausted. The following criteria were applied: (1) no history of previous lumbar surgery, (2) severe low back and leg pain, and no improvement with conservative therapy for at least 6 months, (3) fusion length ≤3 intervertebral segments, (4) spondylolytic spondylolisthesis or spinal deformities, or both, were excluded (viz., if the patient had a coronal curve >30° or a kyphosis >20°). The demographic details of the patients are shown in Table 2.

We included 102 consecutive patients with degenerative spondylolisthesis grade I and II treated at a single institution by two board certified spinal surgeons who have gained expertise in the XLIF procedure before beginning of the study. From April 2012 to March 2014, 56 consecutive patients underwent open PLIF, and from April 2014 to March 2016, 46 consecutive patients underwent single-staged treatment with XLIF, with posterior PPS fixation and intraoperative CT (O-arm) image-guidance navigation as previously described [13] without posterior decompression, and followed up for a minimum of 1 year in the outpatient clinic. Local autologous bone was used in all PLIF PEEK implants and allograft bone was used in all of our XLIF PEEK implants. Resection of rib or iliac bones for bone graft was not performed in the XLIF/PPS group. Patients were allowed to resume activities of daily living the next day depending on their pain from surgery.

### Clinical evaluation

Preoperative and postoperative baseline patient health status were evaluated (for pain-related factors) using the Roland–Morris Disability Questionnaire (RDQ), Oswestry Disability Index (ODI) measured on a 50-point scale, Japanese Orthopaedic Association (JOA) score [14], and the visual analog scale (VAS) score for the lumbar spine at the time of hospital admission and 1 year after surgery.

On postoperative days 1, 4, and 7, serum creatine kinase (CK) and C-reactive protein (CRP), and white blood cell (WBC) counts were measured. The postoperative pain regimen for all patients included a daily dose of celecoxib (200 mg) for the duration of admission. Use of any analgesic regimens except celecoxib was an exclusion criterion for this study. On postoperative day 1, all patients were asked to state their level of pain using a 10 cm VAS with 10 cm indicating the worst pain imaginable. Additionally, on postoperative days 2 through 7, all patients were asked to state their level of pain using a numerical rating scale (NRS) ranging from 0 to 10, with 0 indicating no pain and 10 indicating the pain of surgery on the first postoperative day. On postoperative days 1 through 7, a physiotherapist recorded the performance status (PS) for all patients established by the Eastern Cooperative Oncology Group (ECOG). All personnel involved with the study patients during admission, including the nursing staff and physiotherapists, were blinded to the approach used and objectives of the study. All adverse events during and after surgery were reported. The total perioperative blood loss was estimated as the total of the intraoperative record and drainage output.

### Radiographic evaluation

Preoperative slip (%) of fused levels was evaluated using lateral X-ray images obtained with the patients in a free-standing posture. Bony fusion was assessed by 2 independent physicians using 3-dimensional computed tomography (CT) at 1 year postoperatively, with the grading of fusion classified according to the system described by Bridwell et al. [15] (Table 1).

**Table 1:** Radiological Evaluation with the Bridwell Anterior Fusion Grading System

Grade	Description
1	Fused with remodeling and trabeculae present
2	Graft intact, not fully remodeled and incorporated, but no lucency present
3	Graft intact, potential lucency present at top and bottom of graft
4	Fusion absent with collapse/resorption of graft

### Statistical analyses

Data were analyzed using the unpaired *T* test, Mann–Whitney *U* test and Fisher exact test to determine significant differences. All statistical calculations were performed using Prism (version 6.0; Graph Pad Software, La Jolla, CA, USA). For all tests, *P* < 0.05 was considered significant.

## Results

### Comparison of patient demographics

There were no drop out cases and no revision surgery was needed because of implant failures or adjacent segment disease in either group at 1 year follow-up.

**Fig. 1:**
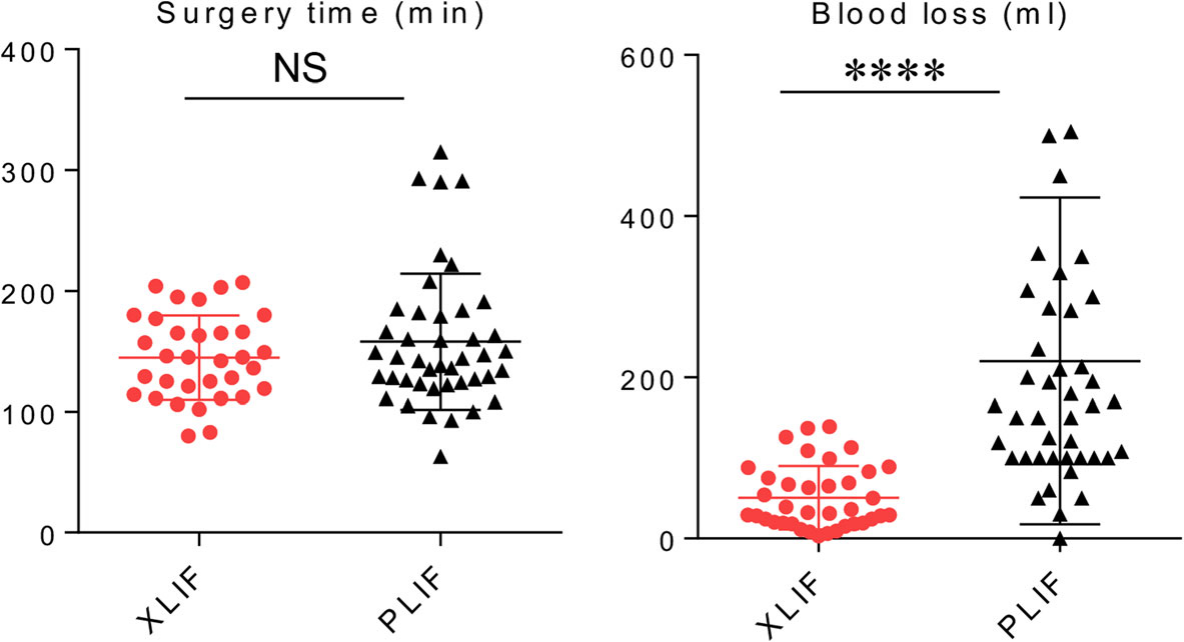
Surgical time and blood loss between XLIF/PPS and PLIF approaches. ****P < 0.0001, NS = not significant. Data were analyzed using the unpaired T test

Table 2 summarizes the preoperative baseline characteristics of the patients who underwent spinal interbody fusion with XLIF/PPS or open PLIF. There was no significant difference in the mean age, the average body mass index (BMI), preoperative slip (%) of fused level, number of fused levels per patient, or proportion of current smokers between the groups (Table 2). The preoperative lumbar–JOA (L-JOA) scores were 14.1 ± 4.5 and 13.5 ± 3.8 in patients in the XLIF/PPS and PLIF groups, and the preoperative ODI scores were 21.2 ± 6.9 and 19.2 ± 6.5, respectively. The preoperative RDQ scores were similar (Table 2). These findings indicated XLIF/PPS and PILF were performed for patients who had similar pain-related parameters. Surgical time was not significantly different between the groups. Estimated blood loss in patients in the XLIF/PPS group was significantly lower than in the PLIF group (51 ± 41 ml in the XLIF/PPS group and 206 ± 191 ml in the PLIF group; *P* < 0.0001) (Fig. 1).

**Table 2:** Demographics of patients undergoing XLIF with PPS or open PILF

	Intraoperative Technique		*P*
	XLIF/PPS (*n* = 46)	PLIF (*n* = 56)	
Age,* y	71.3 ± 8.6	69.0 ± 9.2	0.19
Sex, female/male	31/15	29/27	0.16
BMI,* kg/m^2^	23.4 ± 4.1	23.4 ± 4.6	0.98
Preoperative %Slip,* % Number of fused levels,*	1.88 ± 0.7	1.62 ± 0.8	0.1
Current smoking,* *n* (%)	4 (8.7%)	7 (12.5)	0.75
Preoperative score			
VAS score (lumbar)	4.9 ± 3.2	6.7 ± 2.5	0.37
RDQ score	13.9 ± 5.5	12.8 ± 4.2	0.49
ODI score	21.2 ± 6.9	19.2 ± 6.5	0.17
L-JOA score	14.1 ± 4.5	13.5 ± 3.8	0.41

*XLIF* = extreme lateral interbody fusion, *PPS* = percutaneous pedicle screws, *PLIF* = posterior lumbar interbody fusion, *BMI* = body mass index, *n* = number in group, *VSA* = visual analog scale, *RDQ* = Roland–Morris Disability Questionnaire, *ODI* = Oswestry Disability Index, *L-JOA* = lumbar--Japanese Orthopaedic Association, *Mean ± standard deviation (SD)

### Comparison of serum markers for muscle damage and inflammation

**Fig. 2:**
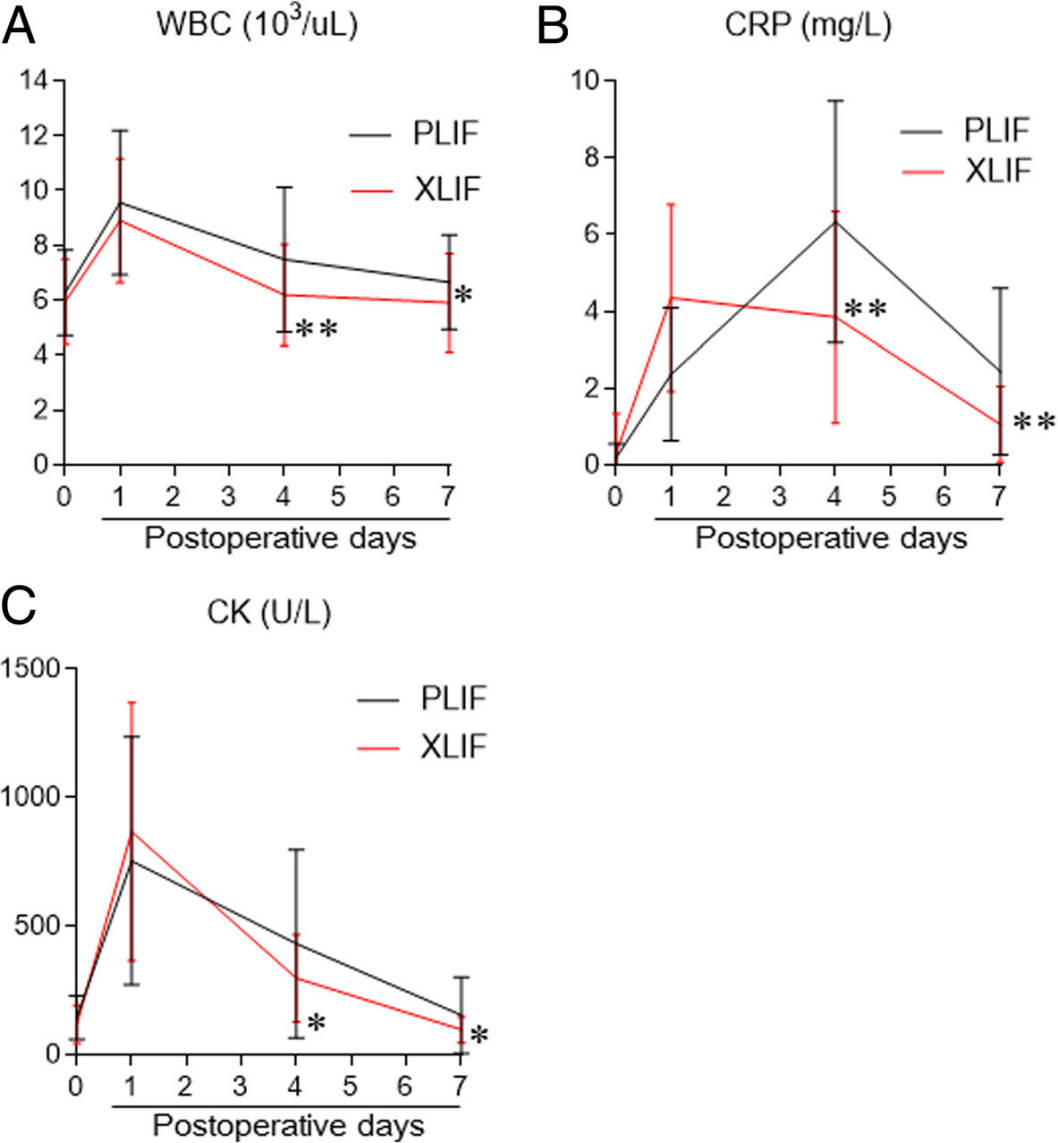
Postoperative serum levels of **a** white blood cells (WBC), **b** C-reactive protein (CRP), and **c** creatinine kinase (CK). *P < 0.05, **P < 0.005. Data were analyzed using the unpaired T test

The postoperative WBC counts and CRP levels were significantly lower in patients in the XLIF/PPS group on postoperative days 4 and 7 (Fig. 2a and b). The postoperative CK levels reached a maximum on the first postoperative day, and there was no significant difference between groups, being 866 ± 503 U/L in patients in the XLIF/PPS group and 753 ± 482 U/L in patients in the PLIF group. Postoperative CK values were significantly lower in patients in the XLIF/PPS group on postoperative day 4 (296 ± 171 U/L in the XLIF/PPS and 430 ± 367 U/L in the PLIF group; *P* = 0.039) and day 7 (93 ± 46 U/L in the XLIF/PPS group and 151 ± 147 U/L in the PLIF group; *P* = 0.025) (Fig. 2c).

### The VAS score and NRS score for surgical pain

**Fig. 3:**
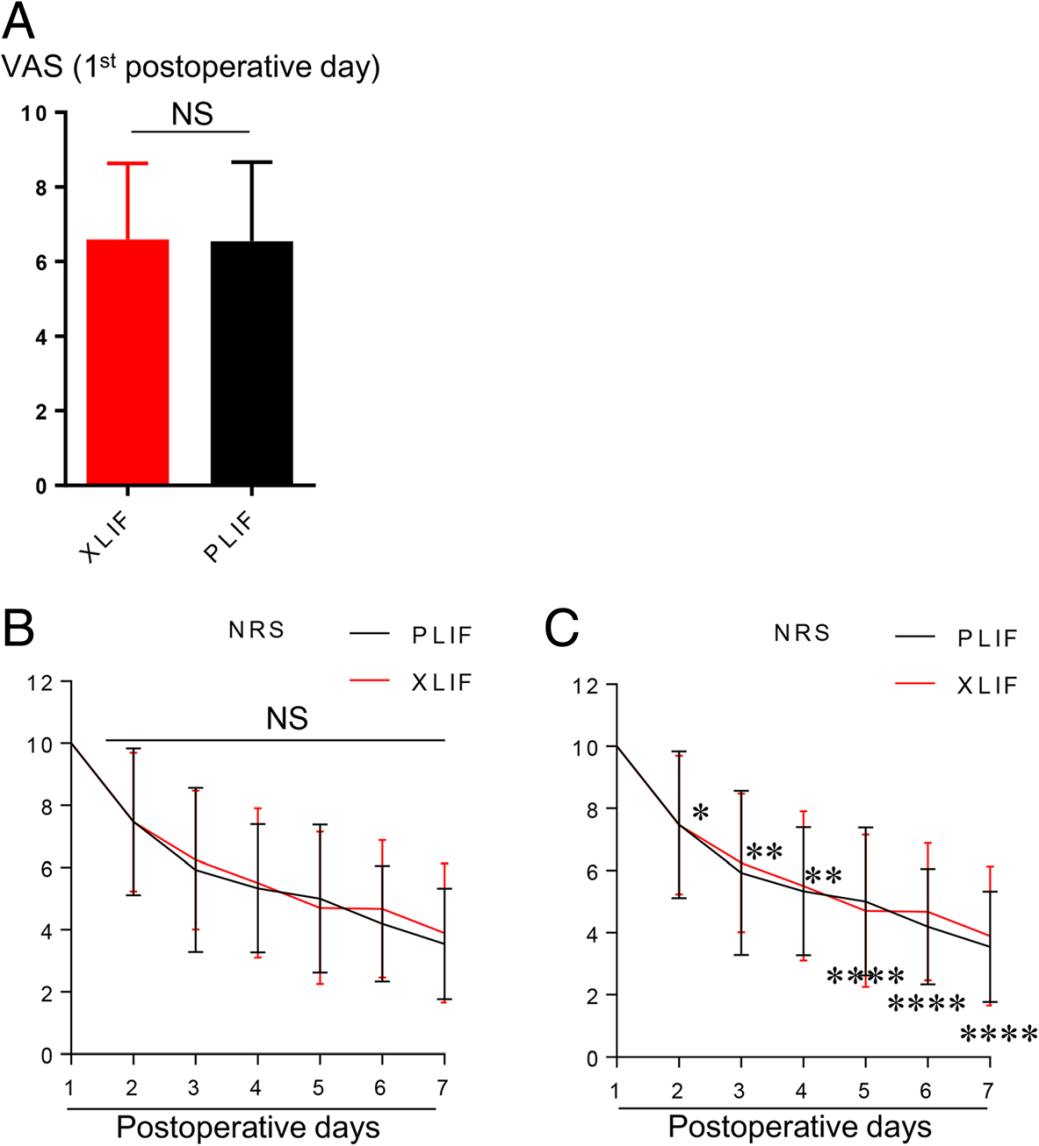
Postoperative **a** VAS score, **b** numerical rating scale (NRS) score, and **c** performance score (PS).*P < 0.05, **P < 0.005, ****P < 0.0001, NS = not significant. Data were analyzed using the unpaired T test for (**a**). Data were analyzed using Mann–Whitney *U* test for (**b**) and (**c**)

The postoperative surgical pain (VAS score) on day 1 was 6.7 ± 2.2 and 6.9 ± 2.3 for the XLIF/PPS and PLIF groups respectively, with no difference between the groups (Fig. 3a). Additionally, there were no significant differences in NRS score for surgical pain between the groups from postoperative day 2 to 7 (Fig. 3b).

### Postoperative recovery of activities of daily living

Postoperative PS scores were significantly greater in the XLIF/PPS group than in the PLIF group from postoperative day 2 to day 7 (Fig. 3c).

### Complications

The surgery-related complications encountered in our study (8.6%) were minor and acceptable (XLIF/PPS group, 6 patients; PLIF group, 2 patients). There were 5 patients who showed a temporary thigh sensory change and 4 patients who showed a temporary hip flexion weakness in the XLIF/PPS group. There was 1 patient who showed superficial disturbance of wound healing and 1 patient in the PLIF group required repair for durotomy. None of the patients in either group required reoperation for surgical site infection, inadequate decompression or instability at the operative levels.

### Comparison of patient outcomes 1 year after surgery

Table 3 summarizes the 1 year postoperative outcomes of patients who underwent spinal interbody fusion with XLIF/PPS or open PLIF. There was no significant difference in the L-JOA score or RDQ between the groups (Table 3). By contrast, ODI and VAS scores (lumbar) 1 year after surgery were significantly lower in the XLIF/PPS group than in the PLIF group. There were no cases of nonunion (grade 3 or 4) in either group and there were no significant differences in the fusion grading between groups using CT at 1 year follow-up (Table 3).

**Table 3:** One-year-postoperative outcomes of patients undergoing XLIF with PPS or open PILF

	Intraoperative Technique		*P*
	XLIF/PPS (*n* = 46)	PLIF (*n* = 56)	
Length of follow-up (years)	2.2 ± 1.2	4.3 ± 2.2	
VAS score (lumbar)	1.5 ± 2.6	3.7 ± 3.1	**<0.005**
RDQ score	8.2 ± 5.4	8.6 ± 5.9	0.95
ODI score	9.2 ± 7.4	13.5 ± 6.4	**<0.05**
L-JOA score	25.3 ± 3.9	24.1 ± 2.4	0.29
Fusion grade	1.5±0.51	1.5±0.5	0.84

*XLIF* = extreme lateral interbody fusion, *PPS* = percutaneous pedicle screws, *PLIF* = posterior lumbar interbody fusion, *n* = number in group, *VSA* = visual analog scale, *RDQ* = Roland–Morris Disability Questionnaire, *ODI* = Oswestry Disability Index, *L-JOA* = Lumbar--Japanese Orthopaedic Association, *Mean ± standard deviation (SD)

## Discussion

Degenerative lumbar spondylolisthesis is one of the disorders most responsive to lumbar fusion. Because of the ability of the interbody fusion technique to correct the listhesis through realignment and stabilization, high rates of improvement on multiple clinical outcome measures after surgery for degenerative lumbar spondylolisthesis have been reported [10]. Recent reports have indicated the effectiveness of XLIF as a surgical treatment for adult spinal deformity, which included greater coronal and sagittal balance correction, and minimized reoperation rate and blood loss [16–18]. By contrast, the benefits of XLIF as a minimally invasive lumbar spinal fusion technique for degenerative lumbar spondylolisthesis remained unclear. Most studies have used comparative approaches focusing simply on complication rates, blood loss, and length of hospital stay as surgical outcomes [11]. Additionally, recent reviews concluded there was insufficient evidence for the effectiveness of XLIF in minimally invasive lumbar spinal fusion and that further studies in support of XLIF in comparison with traditional lumbar interbody fusion approaches are warranted [10, 11]. Therefore, the present study sought to compare XLIF/PPS with traditional open PLIF surgery using multiple outcomes including muscle damage, surgical blood loss, markers of postoperative inflammation, surgical site pain, and postoperative recovery of activities of daily living.

Open PLIF with instrumentation requires extensive soft tissue and back muscle dissection, which is considered to be problematic in the procedure for conventional lumbar fusion [19, 20]. Indeed, a study demonstrated that PPS fixation caused less paraspinal muscle damage than open pedicle screw fixation and had positive effects on postoperative trunk muscle performance [21]. Because it is difficult to quantify blood loss precisely in a minimally invasive procedure, we estimated blood loss as the total of the intraoperative record and drainage output, and found that the XLIF/PPS procedure is extremely advantageous to minimize blood loss. The use of serum markers for inflammation and muscle damage offers objective measures of the invasiveness of the procedure. A postoperative rise in serum CK should indicate the level of muscle damage, and a rise in WBC count and serum CRP levels should indicate the level of inflammation [22]. Our findings showed that WBC counts, and serum CRP and CK levels decreased more quickly in patients in the XLIF/PPS group than in patients in the PLIF group. In accordance with this finding, ODI and VAS score (lumbar) 1 year after surgery were significantly lower in the XLIF/PPS group than in the PLIF group. By contrast, there was no significant difference in the RDQ or L-JOA scores between the groups 1 year after surgery. This finding indicates that although both procedures improved multiple clinical outcome measures compared with PLIF, XLIF/PPS can significantly reduce paraspinal muscle injury which was indicated as less blood loss and lower serum CK level and the incidence of low back pain after surgery.

Unexpectedly, we did not find any difference in postsurgical pain from postoperative days 1 to 7 between the groups in the present study. By contrast, the postoperative recovery of activities of daily living (PS) in patients in the XLIF/PPS group was significantly greater than that in patients in the PLIF group from postoperative days 3 to 7. These findings may result from the difficulty and limitations of accurate self-reported acute pain evaluation using simple pain rating scales [23, 24]. Despite the higher complication rate in the XLIF/PPS group compared with the PLIF group observed in the current study, all of the complications were minor and acceptable. All postoperative thigh symptoms of patients in the XLIF/PPS group were resolved by 1 year after surgery.

This study has limitation that requires further investigation. There was a difference between the two groups in using allograft or autologous bone. This difference could strongly influence postoperative pain, serum creatine kinase and inflammation markers.

However, to our knowledge, this is the first study to indicate the comparative invasiveness and tolerability of XLIF compared with traditional open PLIF as a minimally invasive lumbar spinal fusion method to treat degenerative lumbar spinal disease; not only by surgical blood loss and complication rates, but also by evaluating muscle damage, surgical pain, postoperative recovery of daily activities (performance status score), and incidence of low back pain 1 year after surgery. The XLIF/PPS procedure is advantageous to minimize blood loss and muscle damage with consequent earlier recovery of daily activities (performance status) and a lower incidence of low back pain after surgery, but does not result in less surgical site pain than the open PLIF procedure.

## Conclusions

The XLIF/PPS procedure is advantageous to minimize blood loss and muscle damage, with consequent earlier recovery of daily activities and reduced incidence of low back pain after surgery than with open PLIF.

## Abbreviations

CK: Serum creatine kinase
CRP: C-reactive protein
LBP: Low back pain
NRS: Numerical rating scale
ODI: Oswestry Disability Index
PLIF: Posterior lumbar interbody fusion
PPS: Percutaneous pedicle screws
PS: Performance status
RDQ: Roland–Morris Disability Questionnaire
WBC: White blood cell
XLIF: Extreme lateral interbody fusion

## References

[1] Frymoyer JW, Cats-Baril WL (1991). An overview of the incidences and costs of low back pain. Orthop Clin North Am.

[2] Quint U, Wilke HJ, Löer F, Claes L (1998). Laminectomy and functional impairment of the lumbar spine: the importance of muscle forces in flexible and rigid instrumented stabilization – a biomechanical study in vitro. Eur Spine J.

[3] Quint U, Wilke HJ, Shirazi-Adl A, Parnianpour M, Loer F, Claes LE (1998). Importance of the intersegmental trunk muscles for the stability of the lumbar spine. A biomechanical study in vitro. Spine (Phila Pa 1976).

[4] Sasso RC, Kitchel SH, Dawson EG (2004). A prospective, randomized controlled clinical trial of anterior lumbar interbody fusion using a titanium cylindrical threaded fusion device. Spine (Phila Pa 1976).

[5] O’Toole JE, Eichholz KM, Fessler RG (2009). Surgical site infection rates after minimally invasive spinal surgery. J Neurosurg Spine.

[6] Ozgur BM, Aryan HE, Pimenta L, Taylor WR (2006). Extreme Lateral Interbody Fusion (XLIF): a novel surgical technique for anterior lumbar interbody fusion. Spine J.

[7] Khajavi K, Shen A, Lagina M, Hutchison A (2015). Comparison of clinical outcomes following minimally invasive lateral interbody fusion stratified by preoperative diagnosis. Eur Spine J.

[8] Dickerman RD, East JW, Winters K, Tackett J, Hajovsky-Pietla A (2009). Anterior and posterior lumbar interbody fusion with percutaneous pedicle screws: comparison to muscle damage and minimally invasive techniques. Spine (Phila Pa 1976).

[9] Blizzard DJ, Hills CP, Isaacs RE, Brown CR (2015). Extreme lateral interbody fusion with posterior instrumentation for spondylodiscitis. J Clin Neurosci.

[10] Youssef JA, McAfee PC, Patty CA, Raley E, DeBauche S, Shucosky E, Chotikul L (2010). Minimally invasive surgery: lateral approach interbody fusion: results and review. Spine (Phila Pa 1976).

[11] Barbagallo GM, Albanese V, Raich AL, Dettori JR, Sherry N, Balsano M (2014). Lumbar Lateral Interbody Fusion (LLIF): comparative effectiveness and safety versus PLIF/TLIF and predictive factors affecting LLIF outcome. Evid Based Spine Care J.

[12] Joseph JR, Smith BW, La Marca F, Park P (2015). Comparison of complication rates of minimally invasive transforaminal lumbar interbody fusion and lateral lumbar interbody fusion: a systematic review of the literature. Neurosurg Focus.

[13] Ohba T, Ebata S, Fujita K, Sato H, Haro H (2016). Percutaneous pedicle screw placements: accuracy and rates of cranial facet joint violation using conventional fluoroscopy compared with intraoperative three-dimensional computed tomography computer navigation. Eur Spine J.

[14] Fujiwara A, Kobayashi N, Saiki K, Kitagawa T, Tamai K, Saotome K (2003). Association of the Japanese Orthopaedic Association score with the Oswestry disability index, Roland-Morris disability questionnaire, and short-form 36. Spine (Phila Pa 1976).

[15] Bridwell KH, Lenke LG, McEnery KW, Baldus C, Blanke K (1995). Anterior fresh frozen structural allografts in the thoracic and lumbar spine. Do they work if combined with posterior fusion and instrumentation in adult patients with kyphosis or anterior column defects?. Spine (Phila Pa 1976).

[16] Berjano P, Lamartina C (2013). Far lateral approaches (XLIF) in adult scoliosis. Eur Spine J.

[17] Costanzo G, Zoccali C, Maykowski P, Walter CM, Skoch J, Baaj AA (2014). The role of minimally invasive lateral lumbar interbody fusion in sagittal balance correction and spinal deformity. Eur Spine J.

[18] Dangelmajer S, Zadnik PL, Rodriguez ST, Gokaslan ZL, Sciubba DM (2014). Minimally invasive spine surgery for adult degenerative lumbar scoliosis. Neurosurg Focus.

[19] Sihvonen T, Herno A, Paljarvi L, Airaksinen O, Partanen J, Tapaninaho A (1993). Local denervation atrophy of paraspinal muscles in postoperative failed back syndrome. Spine (Phila Pa 1976).

[20] Taylor H, McGregor AH, Medhi-Zadeh S, Richards S, Kahn N, Zadeh JA, Hughes SP (2002). The impact of self-retaining retractors on the paraspinal muscles during posterior spinal surgery. Spine (Phila Pa 1976).

[21] Kim DY, Lee SH, Chung SK, Lee HY (2005). Comparison of multifidus muscle atrophy and trunk extension muscle strength: percutaneous versus open pedicle screw fixation. Spine (Phila Pa 1976).

[22] Mjaaland KE, Kivle K, Svenningsen S, Pripp AH, Nordsletten L (2015). Comparison of markers for muscle damage, inflammation, and pain using minimally invasive direct anterior versus direct lateral approach in total hip arthroplasty: a prospective, randomized, controlled trial. J Orthop Res.

[23] de C Williams AC, Davies HT, Chadury Y (2000). Simple pain rating scales hide complex idiosyncratic meanings. Pain.

[24] Easton RM, Bendinelli C, Sisak K, Enninghorst N, Regan D, Evans J, Balogh ZJ (2012). Recalled pain scores are not reliable after acute trauma. Injury.

